# The Oxidative Modification of Von Willebrand Factor Is Associated with Thrombotic Angiopathies in Diabetes Mellitus

**DOI:** 10.1371/journal.pone.0055396

**Published:** 2013-01-31

**Authors:** Laura Oggianu, Stefano Lancellotti, Dario Pitocco, Francesco Zaccardi, Paola Rizzo, Francesca Martini, Giovanni Ghirlanda, Raimondo De Cristofaro

**Affiliations:** 1 Institute of Internal Medicine and Geriatrics and Haemostasis Research Centre, Catholic University School of Medicine, Rome, Italy; 2 Section of Biology Applied to Human Health, “Rome Tre” University, Rome, Italy; 3 Diabetes Care Unit, Department of Internal Medicine, Catholic University School of Medicine, Rome, Italy; National Cerebral and Cardiovascular Center, Japan

## Abstract

The thrombogenic activity of Von Willebrand factor (VWF) is proportional to its molecular size and inversely related to its proteolysis by ADAMTS-13. Oxidation of VWF severely impairs its proteolysis by the metalloprotease. This study was aimed at assessing in patients with type 1 and type 2 diabetes whether protein carbonyls, marker of oxidative stress, are associated with both the level and oxidation status of VWF as well as with micro- and macroangiopathic complications. Eighty-three diabetic patients (41 type 1 and 42 type 2 diabetic subjects) and their respective eighty-three healthy controls were studied after verifying the availability, through institutional databases, of clinical biochemistry records spanning at least 3 years. VWF and protein carbonyls were measured by immunoassays, whereas VWF multimers were studied by SDS-agarose gel electrophoresis. Type 2 diabetic subjects had higher levels of VWF antigen (VWF:ag), VWF activity (VWF:act) and plasma proteins’ carbonyls compared to both their controls and type 1 diabetic subjects. Moreover, high molecular weight VWF multimers and specific VWF-bound carbonyls were significantly increased in subjects with micro- and macro-angiopathic complications. In both type 1 and type 2 diabetic subjects, ADAMTS-13 activity was in the normal range. In a multivariable analysis, only VWF-bound carbonyls were significantly associated with any form of thrombotic angiopathy in the entire group of T1- and T2 diabetic patients. These data provide first evidence that not only high VWF levels but also its oxidation status and the presence of high molecular weight VWF multimers that are not observed in SDS-agarose gel electrophoresis of normal subjects are associated with thrombotic angiopathies in diabetes mellitus. These findings may help identify diabetic patients particularly at risk for these complications and elucidate a new pathophysiological mechanism of thrombotic angiopathies in this clinical setting.

## Introduction

Diabetes mellitus (DM) is linked to tissues and organ damage through several pathological mechanisms, such as an increased polyol pathway, increased intracellular formation and activity of advanced glycation end-products, activation of protein kinase C isoforms and finally over-activity of the hexosamine pathway. Altogether, these mechanisms were reported to be linked to an upstream overproduction of reactive oxygen (ROS) and nitrogen species (NOS). In particular, in the diabetic micro-circulation this is a direct consequence of intracellular high-glucose levels [1]. On the other hand, in the DM macrovasculature ROS overproduction would stem from increased oxidation of fatty acids, main consequence of insulin resistance [1]. Moreover, hyperglycemia in DM promotes also an impairment of the antioxidant systems, such as glutathione reduced form (GSH) [2]. Oxidative stress is involved in the pathogenesis of endothelial dysfunction (ED), characterized, besides increased vascular stiffness and tone, by the presence of a prothrombotic and antifibrinolytic state [3]. The oxidative stress causes in these proteins formation of ROS- and NOS-modified amino acids, such as 3-nitrotyrosine (3-N-Tyr) and sulfoxy-methionine (Met-SO). The presence of these oxidized amino acids can affect functional properties of these proteins. In particular, both oxidized fibrinogen and VWF show pro-thrombotic tendency [4]–[6]. Moreover, oxidative stress may also induce accumulation of high molecular weight VWF multimers (UL-VWF), although at a minor extent than in thrombotic microangiopathies, where ADAMTS-13 is strongly reduced or absent so that proteolytic processing of UL-VWF multimers is severely defective. Notably, UL-VWF multimers have the highest ability to recruit and activate platelets in the circulation. The formation of Met-SO at position 1606 in the A2 domain of VWF is indeed responsible for severe resistance to proteolysis by ADAMTS-13 [5], [7], thus favouring an accumulation of UL-VWF multimers [5]. Although it is known that oxidative stress is present in subjects with diabetes, it is still debated how this condition is mechanistically linked to micro- and macro-vascular complications in this setting [8]. Thus, this study was aimed at assessing whether: i) the reported oxidative stress in DM involves also VWF, changing its levels and multimeric structure; ii) the proteolytic processing of VWF by ADAMTS-13 is altered by changes of either levels or function of ADAMTS-13; iii) the presence of oxidized VWF is associated with micro- and macro-angiopathic disorders in a set of both type 1 and type 2 diabetic patients.

## Methods

### Subjects

Diabetic subjects (n = 83, 41 with type 1 diabetes (T1-DM), and 42 with type 2 diabetes (T2-DM)) were consecutively recruited from the outpatient diabetes clinic of the “A. Gemelli” hospital at the Catholic University School of Medicine of Rome. Inclusion criteria required that participants were adults (>18 yr old), did not have arrhythmia and abnormal serum electrolyte levels. An essential criterion for inclusion in the study was the availability, through databases, of HbA1c and other clinical biochemistry records spanning at least 3 years. Type 1 diabetes was diagnosed by the WHO guideline and informed written consent was obtained from each participant. No limits of disease duration were adopted to have a fully representative population of type 1 diabetes subjects referring to a diabetes clinic. Recruited subjects with type 1 diabetes had a disease duration ranging from 3 to 42 years (mean 21.4±10.3 yr). Likewise, recruited type 2 diabetes subjects had disease duration of 2–55 years (mean 18±12 yr). The study was approved by the ethics committee of the Catholic University School of Medicine (EC n. A/493/2010, approved on 05/20/2010) and was conducted according to the recommendations of the Declaration of Helsinki. After the participants had given informed consent, we retrieved the sequential measurements of HbA1c and other clinical biochemistry parameters over the preceding 3 years. The main demographic and clinical characteristics of the study subjects are shown in Tables 1–2. All participants underwent a complete physical examination and completed questionnaires for diabetes duration, previous and current diseases, and use of medications. Subjects were all non-smokers. Ex-smokers were considered those who quit smoking for at least 3 years. Body weight, height, and waist circumference were measured in light clothing, and body mass index (BMI) was calculated. Hypertension was defined according to the current ESC/ESH guidelines [9]. The presence of diabetic microangiopathy was assessed by renal and retinal alterations. The former were evaluated by calculation of the glomerular filtration rate (GFR) and microalbuminuria. The estimated GFR was calculated with the MDRD formula [10]. Direct fundoscopy was performed through dilated pupils by an experienced ophthalmologist, following the EURODIAB diabetic retinopathy scale [11]. Macroangiopathy was defined as any well documented case of ischemic heart disease (IHD), stroke and thrombotic peripheral artery disease (PAD) occurred and diagnosed 90±10 days prior to the enrolment visit. The diagnosis of IHD included coronary insufficiency and nonfatal myocardial infarction, while typical effort angina was excluded. All myocardial infarction case patients met the criteria of diagnostic ECG changes alone or two of the following criteria: typical chest pain of ≥20 minutes duration, abnormal troponin T levels at least twice the upper limit of normal, or characteristic ECG changes. Coronary insufficiency was considered if typical retrosternal chest pain of at least 15 minutes duration was associated with transient ischemic ECG changes but without significant elevation of troponin T or creatine-kinase levels. Diagnoses of myocardial infarction and coronary insufficiency were confirmed by hospital charts. The diagnosis of effort angina was based on symptoms of retrosternal squeezing or pressure-type discomfort occurring on exertion and relieved by rest or nitroglycerin. Exclusion criteria were left ventricular ejection fraction <30%, lung or liver failure, and known cause of anemia and thrombocytopenia (recent overt bleeding, congenital or acquired haematological disease, gastrointestinal disorder, and malignancy). Stroke diagnosis was confirmed by hospital charts and validated by tomographic examinations. Mild-to-moderate peripheral arterial disease (PAD) was diagnosed if the ankle brachial index (ABI) ranged from 0.41 to 0.90 or a history of limb revascularization was present. The mean ABI index in these patients was equal to 0.81±0.06. A complete list of drugs taken by each patient was carefully registered. Healthy subjects(n = 41) among blood donors from the institutional blood bank of the “A. Gemelli” hospital of the Catholic University School of Medicine, Rome, Italy were consecutively enrolled as controls for type 1 diabetes patients. They were between 38 and 55 years of age, were in good health, not smokers and had no risk factors for cardiovascular disease. Forty-two healthy, not smoking subjects between 40 and 79 years without signs of cardiovascular disorders were consecutively enrolled as controls for type 2 diabetes patients. The control group 1 and 2 were age- and sex-matched with type 1 and type 2 diabetic patients, respectively. The control groups were also blood group-matched with the patients based on the known effect of blood group on the level of circulating VWF [12].

**Table 1:** Clinical and biochemical characteristics of T1-, T2-DM patients and respective controls.

Parameter	T1 DM	Controls (T1DM)	P*	T2 DM	Controls (T2DM)	P*	P**
n	41	41		42	42		
Age (yrs)	41±14^§^	42±13	0.66	63.8±9.9	59.9±11.9	0.106	**<0.001**
Sex (M/F)	16/19	15/20	0.92	19/23	29/13	0.71	0.456
DM duration (yr)	21±10	null	n.a.	18±12	null	n.a.	0.303
BMI	27.2±2.1	27±2	0.23	**28±4**	**25±1.7**	**<0.001**	0.67
**HbA1c (%)** *	**7.6±1.1**	**5.1±10.4**	**<0.001**	**7.7±1.2**	**5.1±0.4**	**<0.001**	0.134
Cholesterol total (mg/dl)	184±29	179±30	0.49	170±37	167±35	0.704	0.234
Cholesterol (HDL) (mg/dl)	60±11	52±10	0.16	**46±10**	**58±11**	**0.065**	**0.026**
Cholesterol (LDL) (mg/dl)	108±27	106±29	0.55	101±38	103±37	0.6	0.12
Triglycerides (mg/dl)	85±148	83±29	0.87	**133±50**	**86±30**	**<0.001**	**<0.001**
Blood group “0″	37%	37%	n.a.	38%	38%		
“ “non-0”	63%	63%	n.a.	62%	62%	n.a.	0.987

Legend: ^§^The values of mean±SD are listed;

* Comparison with respective controls;

** Comparison between type 1 and type 2 patients. The parameters with significantly different (p<0.05) values are listed in bold.

**Table 2:** Biochemical characteristics and therapy of T1- and T2-DM patients and respective controls.

Parameter	T1 DM	ControlsT1DM	P*	T2 DM	ControlsT2DM	P*	P**
**n**	41	41		42	42		
**Hypertension**	9/41 (22.9%)	null	n.a.	29/42 (59.5%)	Null	n.a.	**<0.001**
**Creatinine (mg/dl)**	**1.04±0.17**	**0.93±0.1**	**0.002**	**2.4±2.3**	**0.9±0.3**	**<0.001**	**<0.001**
Microalbuminuria (mg/L) ***	12±16	6±2	n.a.	**145±240**	**15±10**	**<0.001**	**<0.001**
GFR calculated (ml/m^2^ min)	88**±**16	90**±**18	0.592	**51±25**	**85±16**	**<0.001**	**<0.001**
**Macroangiopathies**	**3/41**			**21/42**			
**(AMI. Stroke, PAD)**	**7%**	null	n.a.	**50%**	null	n.a.	**<0.001**
**Therapy:**							
**Oral hypoglycemic**	**12/41(29%)**	Null	n.a.	**26/42 (62%)**	Null	n.a.	**<0.001**
**Insulin**	**41/41(100%)**	Null	n.a.	**10/42 (24%)**	Null	n.a.	**<0.001**
**ACE inhibitors**	**6/41 (15%)**	Null	n.a.	**21/42 (50%)**	Null	n.a.	**<0.001**
**Statins**	**6/41 (15%)**	Null	n.a.	**23/42 (55%)**	Null	n.a.	**<0.001**
**Aspirin**	**4/41 (10%)**	Null	n.a.	**22/42(52%)**	Null	n.a.	**<0.001**

Legend: ^§^The values of mean±SD are listed;

* Comparison with respective controls;

** Comparison between type 1 and type 2 patients;

*** v.n. <25 mg/L The parameters with significantly different (p<0.05) values are listed in bold.

### Clinical Biochemistry and Haematological Parameters

Analyses of HbA1c, glucose and blood lipids were carried out at the Department of Clinical Biochemistry, A. Gemelli Hospital in Rome. HbA1c was measured in whole blood by ion exchange high performance liquid chromatography. Triglyceride and HDL cholesterol and LDL cholesterol were measured in serum using an Olympus auto-analyzer. Microalbuminuria was measured by nephelometry (Behring Nephelometer, using reagents from Dade Behring Diagnostics, Marburg, Germany). Basic haematological and coagulation parameters were measured using an automatic blood cell cytometer (Sysmex SF-3000, Dasit, Milano, Italy) and ACL TOP coagulometers (Instrumentation Laboratory, Milano, Italy), respectively.

### Carbonyl Group Content of Plasma Proteins and Purified VWF

The protein carbonyl content was measured as a stable biomarker of oxidative modification of proteins. Carbonyl (CO) groups (aldehydes and ketones) are produced on protein side chains (especially of Pro, Arg, Lys, and Thr) when they are oxidized [13]. The plasma protein carbonyl content was measured according to the OxiSelect™ Protein Carbonyl ELISA (Cell Biolabs, Inc.,San Diego, CA, USA), which quantifies carbonyl groups by derivatizing proteins with dinitrophenylhydrazine (DNP). The sensitivity limit of the method was about 10 pmol carbonyls/mg of protein and the intra- and inter-assay CV was equal to 5.4% and 8.7%, respectively. In a subgroup of subjects (28 cases) from type 2 diabetes patients with the highest plasma protein carbonyls level (>200 pmol/mg), it was reliably possible to measure the specific carbonyl content of VWF, purified from plasma according to a previously described method, with some modifications [5]. Frozen plasma from each subject (20 ml) was thawed at room temperature, added with 0.2 g of PEG [poly(ethylene glycol)]-6000 (Sigma) to a final 1% (w/v) concentration, and gently stirred for 15 min. This solution was added with 1 mM PMSF (0.2 ml, 0.1 M) and 10 mM EDTA (0.4 ml, 0.5 M), as protease inhibitors and left overnight at 4°C under gentle magnetic stirring. The suspension was then centrifuged at 3000 rev./min for 1 h at 4°C. In each test tube, the supernatant was discarded and the pellet resuspended under gentle stirring with 0.2 ml of 110 mM sodium citrate buffer, pH 7.4, and 0.75 ml of 25 mM Tris/HCl, pH 6.8, containing 0.35 M NaCl and 2.6 M glycine. The suspension was centrifuged at 3000 rpm for 45 min at room temperature. The pellet was discarded, while the supernatant was added with solid NaCl to a final concentration of 1.55 M. The suspension was stirred for 30 min and then centrifuged at 6000 rpm for 30 min at 25°C. The supernatant was discarded, and the pellet (derived from the 20 ml plasma pool) was dissolved in citrate buffer (1 ml), divided into aliquots and stored at −80°C. Finally, the solution was fractionated by size exclusion HPLC (SE-HPLC), using a TSK gel Super SW3000 column equilibrated with 20 mM phosphate buffer, 0.15 M NaCl, pH = 7.40, at a flow rate of 0.2 ml/min. The fractions contained in the void volume were collected and analyzed by SDS/PAGE and western blot, using a polyclonal anti-VWF Ab (Dako). No contaminating protein was observed in the SDS-PAGE gel. The concentration of purified VWF was determined spectrophotometrically by measuring the absorbance value at 280 nm, using a molar absorption coefficient of 0.846 mg^−1^ · cm^2^, calculated on the amino acid sequence of VWF monomer. The quality of VWF preparations was also assessed by measuring VWF concentration as antigen (VWF:Ag) and RiCof (ristocetin cofactor) (VWF:RiCof), according to the immunoturbidometric assays ‘VWF antigen’ and ‘VWF activity’ (Instrumentation Laboratory), as detailed previously [5]. The purified VWF was concentrated with a Sartorius Vivaspin 500 centrifugal disposable ultrafiltration device at 14000 rpm in a Thermo microfuge and its carbonyl content measured as detailed above.

### Measurement of Plasma VWF:ag, VWF:act, and ADAMTS13 Level

Blood samples were collected in 3.8% citrate and rapidly stored at −80°C. VWF:ag in plasma was measured using HemosIL von Willebrand Factor Antigen latex immunoassay kits (HemosIL, Instrumentation Laboratory, Milano, Italy) with ACL TOP coagulation system analyzers (Instrumentation Laboratory, Milano, Italy). VWF activity (VWF:act) was measured with a ristocetin cofactor assay using lyophilized platelets (BC VWF reagent kit, Siemens, Milano, Italy) with a BCS coagulation system analyzer (Siemens). The lower limit of normal level for both VWF:ag and VWF:act is 54 IU/dL. The validated normal range of the VWF:act/VWF:ag ratio (n. 200 normal donors) was from 0.71 to 1.35, with mean at 0.98±0.34. ADAMTS13 protease activity (expressed as percentage of normal) was measured by a fluorescence resonance energy transfer based assay using a VWF86 amino-acid peptide substrate (Instrumentation Laboratory, Milano, Italy) in a Varian Eclipse spectrofluorimeter. ADAMTS-13 antigen was measured by an EIA assay (ADAMTS-13 antigen, Instrumentation Laboratory, Milano, Italy).

### Plasma VWF Multimer Analysis

Plasma VWF multimers were analyzed by discontinuous (0.8–1.2%) SDS-agarose electrophoresis and western blotting, as previously described [5]. Images were acquired and analyzed using a ChemiDoc™ XRS+ system (Bio-Rad, Richmond, CA, USA). The quantitative evaluation of UL-VWFmultimers, was performed using the method proposed by Udvardy et al. [14]. UL-VWF was defined as high molecular weight multimers (>10000 kDa), not observed in multimer pattern of plasma VWF from healthy subject. The identification of these high molecular weight forms was also facilitated by comparing the electrophoretic pattern of recombinant VWF, a generous gift of Dr. Friedrich Scheiflinger (Baxter Innovations GmbH, Vienna, Austria), which does not contain any proteolyzed VWF band and includes multimers with molecular weight >10000 kDa. In particular, the amount of UL-VWFmultimers was expressed with the M_MW_ parameter [14]. Digital images of the membranes were obtained by a ChemiDoc MP calibrated densitometer and processed by its QuantityOne software (Bio-Rad Laboratories, Richmond, CA, USA). The background density of the membrane image was subtracted coarsely in a protein-free area. The resulting density (RD) of each VWF peak against relative mobility (RM) data was used for subsequent computations. The MMW parameter assesses the degree of multimerization. First, a curve of RD against RM values was constructed. The upper 25% of the total area under the densitogram peaks was calculated. The molecular weight corresponding to the lower boundary of the 25% of densitometric area was used to calculate the MMW parameter. The molecular weight corresponding to this RM (M_MW_) was estimated based on the correlation of the VWF peaks mobility and their molecular weight, estimated as 500 kDa for a homodimer unit corresponding to the lowest band at the bottom of the gel in each lane [14].

### Hydrolysis by ADAMTS-13 of VWF Purified from Clinical Samples

Von Willebrand factor, purified as detailed above from clinical samples, was used in these functional experiments. In particular, 2 fractions with the highest VWF-bound carbonyls (380 and 230 pmol/mg, respectively) were pooled. Purified VWF from normal subjects (n = 2, carbonyl = 40 pmol/mg) was also used as a control. The working solution contained VWF preparations at a final concentration of 4 µg/ml and incubated with 5 nM recombinant ADAMTS-13 (final concentration, CHO-derived, gene sequence from Gln34 to Trp688, with a C-terminal 10 His tag, purchased from R&D Sytem, Space Import-Export srl, Milano, Italy) in 1.2 mg/ml ristocetin (sulfate-free, used to unfold VWF under static conditions) [15], 5 mM Tris–HCl, 3 mM CaCl_2_, pH 8.0, at 37°C. An aliquot (50 µl) of this solution was sampled at 0 and 60 min. and the reaction stopped by adding 10 mM EDTA. Hydrolysis of the samples was assessed by SDS–agarose electrophoresis in a 1.5% agarose gel and western blot, as detailed above.

### Statistical Analysis

**Figure 1:**
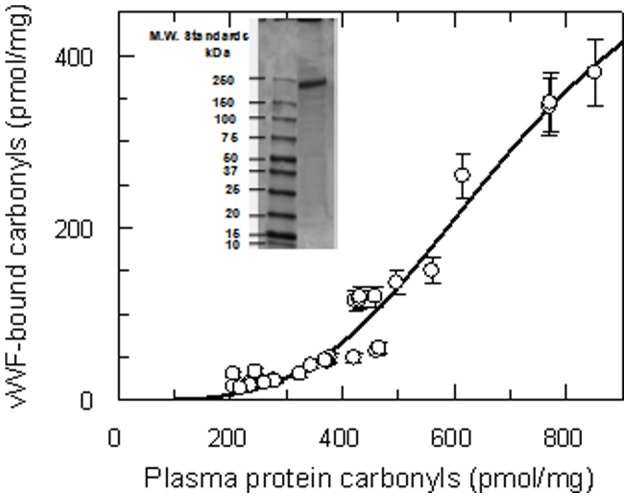
Correlation between total carbonyl content of plasma proteins and that of VWF purified from plasma samples of type 2 diabetes patients. The continuous line was drawn according to this phenomenological equation VWFcarb = (VWFcarb)max x (Pcarb)h/(P50h+Pcarbh), where (VWFcarb)max is the asymptotic value of VWF carbonyls (best fit value: 587±146 pmol/mg), Pcarb is the carbonyl content of plasma proteins, h is an exponential parameter (best fit = 3.6±0.6) and P50 is the concentration of plasma proteins carbonyls equal to (VWFcarb)max/2 (best fit: 706±107 pmol/mg). The vertical bars are the standard errors. In the inset, the SDS-PAGE gel (4–12%) of the purified and reduced VWF preparation is shown. On the left the molecular weight standards are also shown.

All the biochemical parameters contained in the electronic database were monitored for a previous period of 3–5 years. The values pertaining to the biochemical variables (e.g. HbA1c, triglycerides etc.) used in the statistical analyses were expressed as mean one standard deviation of the 4–5 values measured in the year preceding the enrolment visit. The values of the oxidation markers and haemostatic parameters measured during the study and used in the statistical analyses were the mean of two measurements taken in two different occasions over a time interval of seven days, starting from the enrolment visit. In all cases, the two values differed by less than 10%. Previous studies showed that VWF levels are proportional to CV risk and that values >150% are associated with significant odds risk for both acute myocardial infarction and stroke. Assuming a) an expected difference of at least 30% between the mean values of some parameters such as VWF levels and carbonyls pertaining to each class of patients and controls, and b) a standard error of parameters of interest within 10%, we calculated that, enrolling about 40 subject/arm, the potency of the study was >0.95 with α = 0.05. Continuous variables with a normal distribution, according to the Kolmogorov-Smirnov test, were compared by t-test with Welch’s and Bonferroni’s correction to avoid biases arising from unequal variance and Type 1 errors, while skewed ones were compared by Mann-Whitney U test. Categorical variables were compared by Pearson’s chi-square test. Multivariable logistic regression models were used to assess the significance of covariate-adjusted associations between continuous haemostatic and oxidation biomarkers and occurrence of any form of thrombotic angiopathy. Thus, a multivariable logistic backward regression analysis was performed using the data pertaining to both T1- and T2DM patients. In this multivariable model, the occurrence of any vascular complication (micro- or macroangiopathies) was analyzed as a function of fibrinogen, VWF:act and VWF-bound carbonyls. These covariates were age- and sex-adjusted in the analysis. In the final model, total plasma protein carbonyls and D-dimer were excluded as independent variable to avoid the bias of multicollinearity [16], as in univariate analysis their values were correlated with the level of VWF-bound carbonyls (p<0.001, see also Fig. 1) and fibrinogen (p = 0.037). Values of measured variables were reported as mean ± standard deviation, unless otherwise indicated. A two-sided p value <0.05 was required for statistical significance. Analyses were performed using SPSS software (version 13, SPSS, Chicago, IL, USA). Graphpad Prism software (version 5.00 for Windows, GraphPad Software, San Diego, CA, USA) was used to construct appropriate graphs.

## Results

### Clinical and Biochemical Characteristics of Type 1 and Type 2 Diabetic Patients

The main characteristics of both T1-DM and T2-DM patients compared to respective controls are listed in Tables 1, 2, 3. In particular, only HbA1c in T1-DM subjects had a significantly higher value than in control subjects (see Table 1). The activity of ADAMTS-13 although significantly lower than in control subjects (83% vs 110.7%), was in the normal range (see Table 3). On the contrary, T2-DM subjects, though having comparable disease duration compared to T1-DM patients, showed higher levels of HbA1c, BMI, triglycerides and creatinine with lower glomerular filtration rates (see Tables 1–2). Likewise, coagulation parameters and the protein oxidation biomarker (fibrinogen, VWF:activity, VWF:antigen, d-dimers and protein carbonyls) were higher in T2-DM subjects than in the relative controls and T1-DM patients (see Table 3). T2-DM patients, like T1-DM subjects, had ADAMTS-13 activity in the normal range (94.9±37.1, Table 3). However, the ADAMTS-13/VWF:act ratio was lower in both T1- and T2-DM patients compared with their respective controls, whereas no statistically significant difference was observed between the two groups of diabetic patients (see Table 3). The decreased ADAMTS-13/VWF:act ratio is not of unequivocal interpretation in the presence of normal levels of both ADAMTS-13 and VWF, although the decrease of this derived parameter might reflect a situation less favorable to an efficient ADAMTS-13/VWF interaction. APTT and prothrombin time were in the normal range for both T1- and T2-DM patients. It has to be remarked that treatment with aspirin (100 mg/day) and statins was applied in 52% and 55% of T2-DM subjects, respectively (see Table 2). Notably, all patients with previous macro- and diffuse micro-angiopathy (retinopathy plus renal impairment) were under treatment with aspirin.

**Table 3:** Haemostatic and oxidative biomarker levels in T1- and T2-DM patients and respective controls.

Patameter	T1DM	Controls T1DM	P*	T2 DM	Controls T2DM	P*	P**
n	41	41		42	42		
Fibrinogen (mg/dl)	250±31§	245±34	0.546	**352±118**	**264±50**	**<0.001**	**0.0169**
D-dimer (ng/ml)	196**±**187	160**±**57	0.289	**306±243**	**171±59**	**<0.001**	**0.0008**
VWF:ag (%)	110±42	108±24	0.789	**165±65**	**115±26**	**<0.001**	**<0.001**
VWF:act (%)	108**±**41	97**±**18	0.156	**148±72**	**102±22**	**<0.001**	**0.0056**
VWF-R (Act/Ag)	0.92**±**0.2	0.91**±**0.1	0.051	0.94±0.5	0.9**±**0.13	0.618	0.589
ADAMTS13 Antigen (ng/ml)	595**±**157	629**±**143	0.292	592**±**198	589**±**185	0.936	0.976
**ADAMTS13 Activity (%)**	**83±21**	**111±25**	**<0.001**	95**±**37	100**±**32	0.8243	0.332
**ADAMTS13/VWF:act ratio**	**0.8±0.5**	**1.14±0.5**	**0.038**	**0.6±0.5**	**0.98±0.5**	**0.021**	0.214
Log MMW	6.1**±**0.7	6.1±0.6	0.63	**7.3±0.8**	**6.2±0.7**	**0.0062**	**0.0065**
Protein carbonyls (pmol/mg)	167**±**9	140±13	0.35	**340±170**	**128±7**	**<0.001**	**<0.001**

* Comparison with respective controls;

** Comparison between type 1 and type 2 patients.

§ The values of mean±SD are listed in the table.

### Univariate Analyses of Clinical, Biochemical and Haemostatic Variables Measured in Diabetic and Control Subjects

Individual results of univariate analysis of clinical, biochemical and haemostatic variables measured in all diabetics and control subjects showed that the level of plasma protein carbonyls was significantly and positively correlated with age (p = 0.039), APTT (p<0.001), fibrinogen (p = 0.002), VWF:ag (p<0.001), VWF:act (p = 0.003) and any form of micro- (p<0.001) and macroangiopathy (p<0.001). The strong association between carbonyls and VWF levels with micro- and macroangiopathies prompted us to investigate the specific content of carbonyls in VWF. The removal capacity in the chromatographic procedure of VWF purification was equal to 4.43 log_10_. As shown in Figure 1, the carbonyl content of VWF purified from T2DM patients was positively and logistically correlated with the overall carbonyl content of the plasma proteins contained in the same sample. Thus, although VWF is a minor component of plasma proteins, nevertheless it is sensitive to oxidative stress and undergoes oxidative modifications as in the case of much more abundant proteins, such as albumin and fibrinogen [4]. The non linear equation used to analyze the relationship between plasma protein carbonyls and VWF-bound carbonyls (see legend to Fig. 1) allowed also to calculate the latter for all T2DM samples.

### Comparison between Carbonyl Content of Plasma Proteins, VWF and ADAMTS-13 in Diabetic Patients with and without Micro- and Macroangiopathic Complications

**Figure 2:**
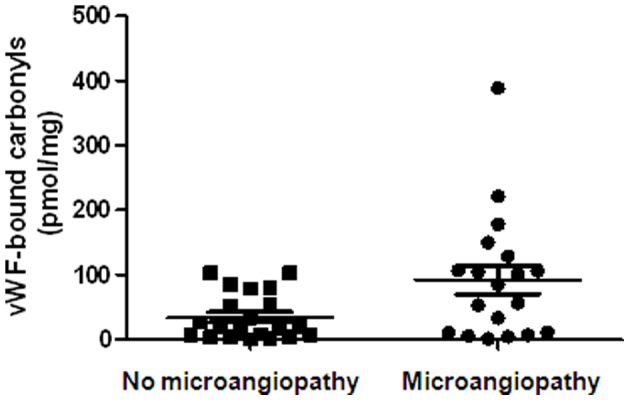
Statistical comparison between calculated VWF-bound-carbonyl levels in type 2 diabetic patients with and without microangiopathic complications (renal and retinal). According to Mann-Whitney test, VWF-bound carbonyls had higher values in microangiopathic subjects than in non macroangiopathic diabetics (mean values ± SD: 92±22 vs 35±8 pmol/mg, respectively, p = 0.022).

**Figure 3:**
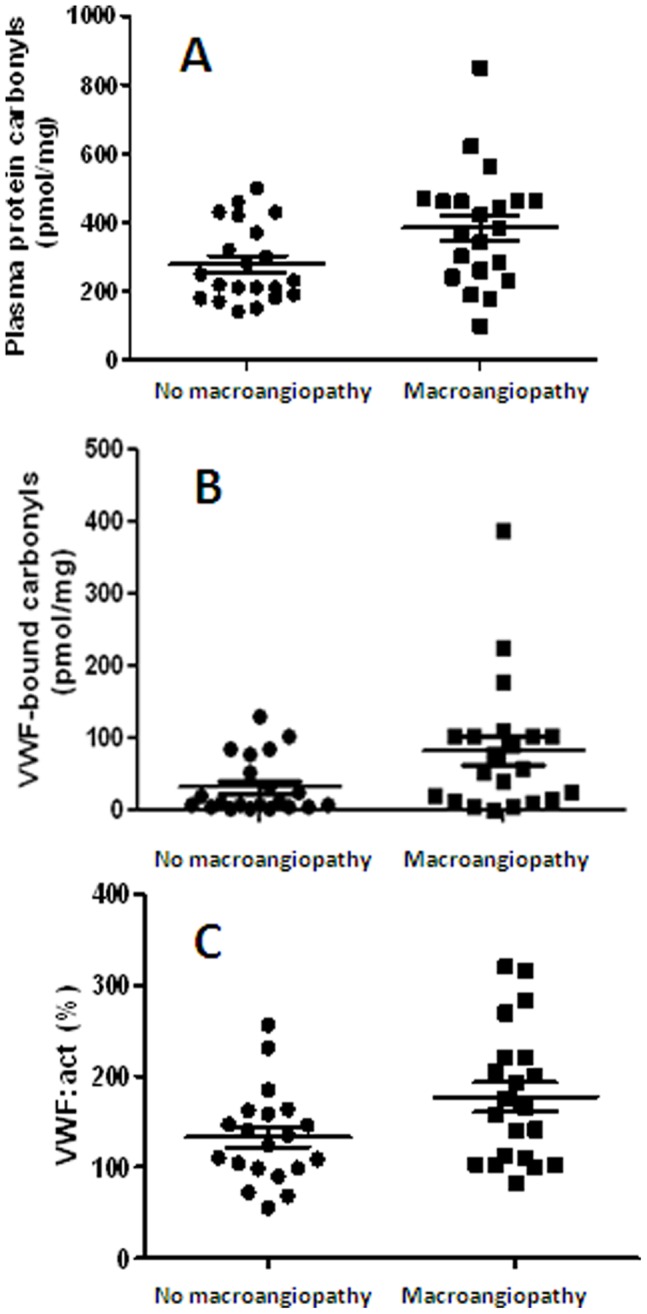
Comparison of total carbonyl content of plasma proteins, VWF-bound carbonyls and VWF:act between type 2 diabetic patients with and without macroangiopathies (AMI, stroke and PAD). According to Mann-Whitney test, all these parameters showed higher values in macroangiopathics than in non macroangiopathic diabetics (mean values ± SD: A) total carbonyl content of plasma proteins, 380±37 pmol/mg vs 270±25 pmol/mg, p = 0.0234; B) VWF-bound carbonyls, 82±20 vs 33±9 pmol/mg, p = 0.028; C) and 177±16% vs 132±12%, p = 0.032, respectively.

In T2-DM with microangiopathic complications (either renal or retinal or both), only total plasma protein carbonyls (384±170 pmol/mg vs 270±110 pmol/mg p = 0.023) and VWF-bound carbonyls (92±20 vs 35±8 pmol/mg, p = 0.022) were found significantly increased compared with not microangiopathic patients (Figure 2). On the other hand, in T2-DM patients with thrombotic macroangiopathies VWF:act, plasma protein carbonyls and VWF-bound carbonyls were significantly higher than in patients not affected from these complications (p = 0.023, 0.032 and 0.028, respectively; see Figure 3). Notably, although the values of fibrinogen was higher in T2-DM than in both controls and T1-DM subjects, no difference was found for this parameter between patients with macroangiopathies compared to diabetics without these complications (p = 0.98). Finally, Table 4 reports the values of haemostatic and oxidation biomarkers of T2DM patients with and without any form (micro- and/or macro-) of angiopathy. Globally, these results show that in angiopathic diabetics, UL-VWF multimers are more expressed and that their oxidative modifications are higher than in not angiopathic patients. On this basis, a multivariate logistic backward regression analysis was performed using the data pertaining to both T1- and T2DM patients. In the final model, VWF-bound carbonyl level, adjusted for age and sex, was the only variable significantly associated with the development of any form of vascular complication in diabetic patients (OR = 28.2, 5–95% CI = 1.2–658, p = 0.038, see Table 5). Instead, no significant association was found for VWF:act and fibrinogen levels (p = 0.511 and 0.362, respectively).

**Table 4:** Haemostatic and oxidative biomarker parameters§ of T2-DM patients with and without any form of angiopathies.

Parameter	No angiopathies	With angiopathies	P (two-tailed)
Fibrinogen (mg/dl)	251**±**77§	392**±**107	0.0023
D-dimer (ng/ml)	227**±**133	378**±**297	0.043
VWF:ag (%)	145**±**54	182**±**71	0.061
VWF:act (%)	132**±**75	193**±**68	0.022
ADAMTS-13 activity (%)	66±34	111**±**28	0.0005
Log MMW	6.1**±**0.6	7.4±1.3	0.0002
Protein carbonyls (pmol/mg)	312±80	370±100	0.035
VWF-bound carbonyls (pmol/mg)	182**±**134	364**±**205	0.002

§ The values of mean±SD are listed in the table.

**Table 5:** Multivariable-adjusted* logistic regression for thrombotic angiopathies with haemostatic and oxidation variables in T1– and T2DM patients (n = 83).

Variables	OR (5–95% confidence interval)	P
VWF-bound carbonyls	28.2 (1.2–658)	0.038
VWF:act	1.003 (0.995–1.011)	0.511
Fibrinogen	1.003 (0.997–1.008)	0.362

* Adjusted for age and sex.

### Association of Plasma Protein Carbonyls with Multimer Size in Type 2 Diabetes Patients

**Figure 4:**
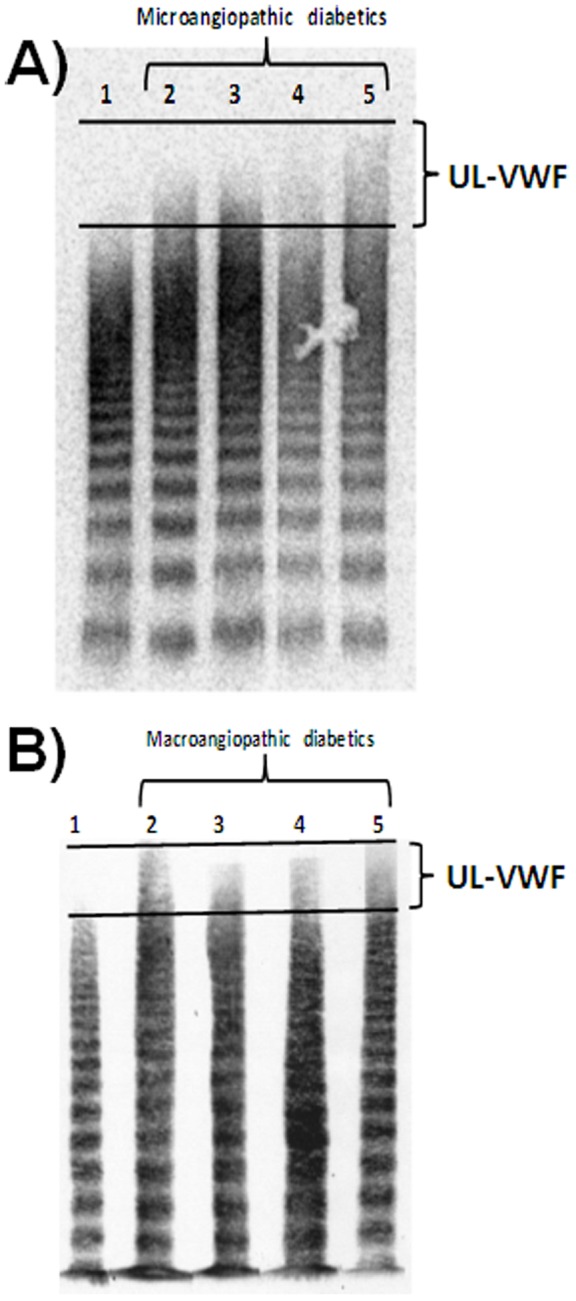
Comparison of SDS-agarose gel electrophoresis and western blot of VWF multimers between type 2 diabetic patients with low VWF-bound carbonyls without microangiopathy and those with high levels of VWF-bound carbonyls and several forms of microangiopathies. A) SDS-agarose gel electrophoresis and western blot of VWF multimers from a pool (n = 10) of type 2 diabetic patients without microangiopathy and low VWF-bound carbonyls (<50 pmol/mg) (1) and with high levels of VWF-bound carbonyls (>100 pmol/mg) and several forms of microangiopathies (2: nefropathy; 3: nefropathy+grade 3 retinopathy; 4: nefropathy+retinopathy+autonomic neuropathy; 5: nefropathy+grade 3 retinopathy; autonomic neuropathy+coronary microangiopathy). The agarose gel was discontinuous (0.4% agarose in stacking gel and 1.2% agarose in running gel. The amount of loaded VWF was similar in all samples (about 2 µg). B) SDS-agarose gel electrophoresis and western blot of VWF multimers from type 2 diabetic patients without macroangiopathy (1) and with several forms of macroangiopathies (2–4: coronaropathy; 5: coronaropathy+PAD). The presence of UL-VWF in samples is indicated between the dashed lines.

**Figure 5:**
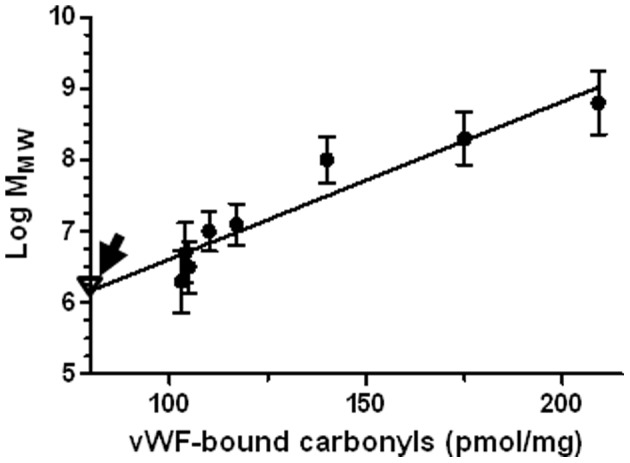
Association of carbonyl content of purified VWF with the MMW parameter for T2-DM patients with macrovascular complications. The continuous line represents the linear regression (R2 = 0.908, p = 0.0002). The arrow shows the mean value of the parameter in age- and sex- matched healthy controls (MMW = 6.2±0.7), all having carbonyl content of VWF<20 pmol/mg (Δ). Notably, all the patients with VWF-bound carbonyls>100 pmol/mg suffered from both micro- and macrovascular complications.

As indicated above, the mean value of plasma protein carbonyls in type 2 DM patients with macroangiopathies was found equal to 309±37 pmol/mg, whereas in patients without macroangiopathies the same parameter value was equal to 270±25 pmol/mg (p = 0.0023). Accordingly, the VWF-bound carbonyl levels were equal to 82±20 pmol/mg in macroangiopathic patients and 33±9 pmol/mg, in not macroangiopathic diabetics (p = 0.028). VWF:act was equal to 177±16% and 132±12% in macroangiopathic and not macroangiopathic patients, respectively (p = 0.032). These findings were in agreement with the hypothesis that VWF of T2-DM patients undergoes oxidative modifications and changes its activity pattern. Hence, we investigated whether or not there was a relationship between the level of VWF-bound carbonyls and the amount of UL-VWF. This was the case, as shown by gels of SDS-agarose electrophoresis of VWF multimers. Figure 4 shows examples of typical cases of T2-DM patients with and without micro-and macro-angiopathies and VWF-bound carbonyls >100 pmol/mg. Under the experimental conditions used in the electrophoretic run, large VWF multimers were not resolved into single bands but appeared as “smear” at the top of the gel. However, this feature was never present in the respective controls and diabetic patients without angiopathic complications, although the loaded VWF amount was the same. A positive linear association was found between VWF-bound carbonyls and the presence of UL-VWF, reflected by the M_MW_ parameter (see Figure 5). Notably, this relationship was found in samples characterized by high carbonyl content of VWF (>100 pmol/mg) and occurrence of severe macrovascular complications. In this subset of T2-DM patients, no statistically significant difference was found between the patients with and without macrovascular complications for age (p = 0.454), disease duration: (p = 0.347) and HbA1c levels (p = 0.658).

### Hydrolysis by ADAMTS-13 of Purified VWF Samples from T2DM and Control Subjects

**Figure 6:**
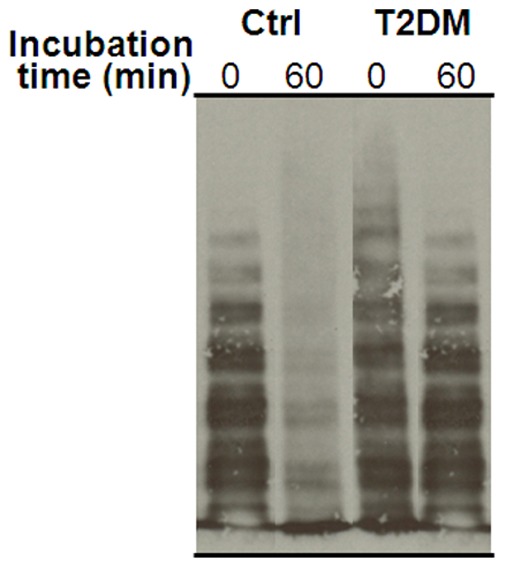
Cleavage by ADAMTS-13 of VWF multimers in a pool obtained from two T2DM patients and two healthy subjects (Ctrl). Purified VWF multimers were digested by 5 nM ADAMTS-13 for 60 min in the presence of 1.2 mg/ml ristocetin under the experimental conditions detailed in the text. The same protein amounts were loaded on the gels. The samples from diabetic patients had the highest VWF carbonyl content (380 pmol/mg), whereas the controls had a lower carbonyl content (40 pmol/mg).

The results of the functional experiments testing the ability of ADAMTS-13 to proteolyze VWF purified from T2DM and control subjects are shown in Fig. 6. The velocity of VWF hydrolysis was significantly lower in the case of VWF factor containing high level of carbonyl group (300 pmol/mg) compared to control VWF containing low amount of carbonyls (40 pmol/mg). After 60 min of incubation, VWF purified from normal controls was extensively hydrolyzed, whereas VWF with high oxidation status from T2DM patients still contained VWF multimers with medium molecular weight.

## Discussion

The main novelty of this study is that not only the level but also the oxidative modification of VWF is strongly associated with both presence of high molecular weight multimers and thrombotic angiopathies in diabetes. Hyperglycemia is known to promote ROS production and impairment of antioxidant systems, such as generation of the reduced form of glutathione (GSH) and vitamin C [8], [17], [18]. Oxidative stress is involved in the pathogenesis of endothelial dysfunction (ED), which promotes a global prothrombotic and antifibrinolytic status [8]. ED plays a central role in the pathogenesis of atherosclerosis and its presence has been documented in patients with diabetes mellitus [19]. The occurrence of ED is associated with the elevation of several markers such as VWF, endothelin-1 (ET-1), vascular endothelial growth factor and vascular cell adhesion molecule-1. In particular, increased ET-1 and VWF levels were found in type 1 and type 2 diabetes patients [20], [21]. Notably, in the Munich General Practitioner Project [22] and in the ARIC study [23], increased levels of VWF:ag were identified as risk factors for macrovascular prevalence and mortality in type 2 diabetes. It is likely that oxidative stress is associated in type 2 diabetic patients with a systemic inflammatory status characterized by increased levels of cytokines as IL6 and TNF-α [24]. These inflammatory cytokines promote the release of VWF from endothelial cells and induce a defect of VWF proteolytic processing by ADAMTS-13 [25]. These effects may cause an increase of both level and size of VWF multimers, expressed by VWF:act. In this investigation only a slight decrease of ADAMTS-13 level was found in diabetics, in agreement with previous reports [26], [27]. However, only in the presence of severe ADAMTS-13 deficiency (level<6%), ultra-large VWF multimers accumulate, causing thrombotic microangiopathies [28]. Large VWF multimers are stored in the Weibel-Palade bodies (WPB) of endothelial cells (ECs) and are released into the bloodstream upon the interaction of several agonists with the respective receptors on ECs. A change in intracellular ROS can activate signal transduction pathways [29], driving in ECs the actin cytoskeleton assembly [30] and WPB mobilization. Hence, the increase of ROS production in diabetes can accelerate the secretion of VWF multimers from endothelial WPB, favouring the release of large VWF multimers (UL-VWF) into the circulation, as indeed shown in this study. The level of carbonyl content of purified VWF was found proportional to the same parameter measured in plasma proteins from diabetic patients. This phenomenon may arise in vivo from the exposure of VWF to oxidizing agents, as peroxynitrite, during the period of storage in the WPB of endothelial cells. In line with this hypothesis, our research group has recently demonstrated the specific oxidation of Met1606 in VWF purified from samples of patients with chronic kidney disease, another setting characterized by high oxidative stress [6]. A significant portion of the type 2 diabetes patients enrolled in this study was affected by diabetic nephropathy (see Table 2). This condition may have contributed to increase the oxidative stress, reflected by high levels of plasma protein carbonyls. However, it has to be remarked that the in our previous study the oxidation of VWF was found elevated in patients with chronic renal failure under hemodialysis [6]. In the present study, the diabetic patients were characterized only by mild or moderate reduction of glomerular filtration rate but none of them was under haemodialysis. Further studies are under way by our research group to investigate the possible presence and effects of oxidative stress on VWF in not diabetic patients with mild/moderate renal failure. Our research group and other investigators have shown that specific oxidation of Met1606 in VWF inhibits its proteolytic processing by ADAMTS-13 [5], [7], [31]. In particular, this effect was demonstrated in VWF purified from plasma of severe T2-DM patients [6].This mechanism may further contribute to favour the accumulation in the circulation of UL-VWF multimers, which have the highest pro-thrombotic activity. Previous studies showed indeed that UL-VWF multimers are present in severe type 2 diabetic patients with very high carbonyl content in VWF [5]. In the present study, we have investigated a larger and more representative diabetic population, in which the presence of UL-VWF multimers was found associated with occurrence of micro- and macro-angiopathic complications. The increased presence of UL-VWFmultimers could not be attributed to ADAMTS-13 deficiency. In this clinical setting, ADAMTS-13 is present at a normal level (see Table 3), sufficient to proteolyze in part UL-VWF multimers, at variance with the situation observed in canonical forms of thrombotic microangiopathies, where ADAMTS-13 is <6%. In a subset of type 2 diabetes patients, we have indeed shown that the setting characterized by VWF-bound carbonyls >50 pmol/mg is significantly associated with micro- and macro-angiopathies. In a multivariate analysis, the level of VWF-bound carbonyls was the only parameter significantly associated in both T1- and T2DM with occurrence of any kind of vascular complication with an OR equal to ≈28 (p = 0.038). This finding supports the hypothesis that beside the level also the oxidation status and the multimeric pattern of this protein are associated with thrombotic vasculopathies. Recently, in an elegant study, Fu et al. have demonstrated that shear stress–induced unfolding of VWF exposes buried, oxidation-sensitive methionine residues, including Met1606, contained in the A1 and A2 VWF domain [32]. Conversion into methionine sulfoxide of these Met residues, buried in the coiled VWF conformation, is strongly facilitated by shear stress–induced unfolding of VWF multimers. Notably, methionine oxidation, besides reduced proteolysis by ADAMTS-13, results also in enhanced VWF binding to GpIb and platelet activation [5], [32]. In addition, basic biophysical principles indicate that the higher the molecular size of the VWF, the higher is the sensitivity to shear stress [33]. Several studies have provided compelling evidence of the relevance of VWF levels in the pathogenesis of macrovascular thrombosis, especially in the brain circulation [34]–[36]. The findings of the present study emphasize also the relevance of the multimeric structure and especially of the oxidation status of VWF multimers for their pro-thrombotic effects. This phenomenon, along with the documented intrinsic hyperactivity of platelets in type 2 diabetic patients [37], [38], can contribute to intensified adhesion, activation, and aggregation of platelets, favoring the occurrence of thrombotic complications in the arterial circulation. VWF has also other extra-haemostatic functions, including angiogenesis and leukocyte extravasation [39], [40], which can cooperate in inducing thrombotic events. The effect of VWF oxidation on these biological functions is still unknown and needs to be investigated to assess its possible contribution to these phenomena. This study has several clinical implications and limits. The results of this pilot study would indicate the need of prospective and intervention studies on diabetic population to correlate the progression of oxidative modification of VWF with major adverse cardiovascular events and their pharmacological prevention. The use of aspirin for the primary prevention of cardiovascular events in diabetic individuals is a widely recommended practice in accordance with existing guidelines [41]. However, several prospective trials have shown that in diabetic patients this drug shows a lower efficacy in protecting against thrombotic macroangiopathies than in not diabetic subjects [42]–[44]. This is likely a multifactorial phenomenon. However, it can be speculated that the lower pharmacological efficacy may be in part linked to lack of specificity of aspirin in inhibiting the VWF-platelet receptor interaction and signaling [45]. The use of novel anti-platelet agents as the inhibitors of the VWF-GpIb interaction [46]–[49] may ameliorate the outcome of antithrombotic therapy in T2-DM patients. Prospective studies are needed to assess whether the association of oxidative stress and VWF abnormalities is only a sustained epiphenomenon of thrombotic diseases or is causally related to macroangiopathic complications in diabetic patients. This conclusion cannot be unequivocally validated by the present study, since it is based on a post hoc analysis. Further work on the specificity and sensitivity of VWF:act and VWF-bound carbonyl level is therefore required to ascertain their role as prognostic biomarkers for thrombotic vasculopathies in type 2 diabetes mellitus. Even without a specific quantification of VWF-bound carbonyls, the measurement of overall level of carbonyls of plasma proteins may be used as a surrogate marker of oxidative modification of VWF, as shown in the present study. In conclusion, the emerging scenario shows that the oxidative stress in diabetes involves also VWF and is associated with increased presence of UL-VWF multimers that are involved in the genesis of major cardiovascular events in this clinical setting.
